# A case of severe flail chest with several dislocated sterno-chondral fractures

**DOI:** 10.1016/j.ijscr.2019.10.043

**Published:** 2019-10-28

**Authors:** Ali Imad El-Akkawi, Frank Vincenzo de Paoli, Gratien Andersen, Anette Højsgaard, Thomas Decker Christensen

**Affiliations:** a Department of Cardiothoracic and Vascular Surgery, Aarhus University Hospital, DK - 8200 Aarhus N, Denmark; b Department of Clinical Medicine, Aarhus University Hospital, DK - 8200 Aarhus N, Denmark; c Department of Biomedicine, Aarhus University, DK - 8000 Aarhus C, Denmark; d Department of Radiology, Aarhus University Hospital, DK - 8200 Aarhus N, Denmark

**Keywords:** Flail chest, Trauma, Chest stabilization, Plate fixation, Cartilage reconstruction, Case report

## Abstract

**Introduction:**

Flail chest is diagnosed clinically by the presence of paradox movement of a segment of the thoracic wall during spontaneous breathing. Radiographic finding confirming a clinical flail chest are fractures of three or more consecutive ribs or costal cartilages in two or more places. Surgical stabilization is associated with a reduced length of hospital stay, time with mechanical ventilation and risk of respiratory complications.

**Presentation of case:**

A trauma patient had a Computed Tomography (CT) scan showing multiple costa fractures, sternal fracture, manubrium fracture, sternal displacement and dehiscence of the sternal-costal attachment. The severity of the trauma was visualized after performing a cartilage reconstruction of the trauma CT scan. The patient underwent surgery, using fixation plates to stabilize the thoracic cage, and was then weaned quickly from mechanical ventilation.

**Discussion:**

This case indicates, that if a patient has a severe flail chest recognized clinically, but not radiologically, a reconstruction of cartilage can reveal the true severity of the trauma. Indeed, the patient in this case experienced a positive outcome from surgery. However, such a procedure demands correct timing and experience in surgical stabilization of the thoracic wall. Furthermore, the injury required accurate planning with the involved personal before surgery.

**Conclusion:**

Surgical stabilization of advanced flail chest with concomitant sternal fracture, seems to be a safe procedure, that might reduce the need of mechanical ventilation and the length of stay at the Intensive Care Unit (ICU). Furthermore, cartilage reconstruction of the trauma CT scan can potentially identify a severe flail chest, that might be missed on regular 3D bone reconstruction.

## Highlights

• Surgical stabilization of flail chest lead to fast weaning from mechanical ventilation.
• Plates can be used for stabilization of sterno-costal dehiscence.
• Cartilage reconstruction of a CT scan can reveal the true severity of the trauma.

## 1 Introduction

Multiple rib fractures and flail chest results of a severe trauma [[1], [2], [3]], and it carries a high morbidity and mortality [2,3]. These patients will most likely undergo a full-body Computed Tomography (CT) scan [4]. When evaluating severe injuries to the thoracic cage a 3D bone reconstruction of the trauma CT scan becomes valuable, and in some cases mandatory. Furthermore, additional 3D reconstruction of the cartilage can reveal injuries that might be missed on a 3D bone reconstruction.

Flail chest is clinically diagnosed by the presence of paradox movement of a segment of the thoracic wall during spontaneous breathing. Radiographic findings are fractures of three or more consecutive ribs or costal cartilages in two or more places [2]. Flail chest is associated with a poor outcome, in terms of prolonged hospital stay, pneumonia, respiratory distress and need of mechanical ventilation.

Flail chest with concomitant sternal fracture occurs in 14% of cases and is associated with an even higher risk of complications and additionally increased mortality [5]. The treatment of flail chest is predominantly conservative, e.g. by analgesia and respiratory support. Surgical treatment has shown good results in terms of a reduced length of hospital stay, time with mechanical ventilation and risk of respiratory complications such as pneumonia and respiratory distress. However, the evidence is primarily based on case reports [6,7] and only a few very small randomized, controlled trials exist [8]. Hitherto, the level of evidence is relatively low.

The evidence regarding the surgical approach in treatment of flail chest with concomitant sternal fracture is low, predominantly due to a low number of cases. The approach might even be different depending on which region on sternum that is fractured and the type and numbers of fractures resulting in flail chest [9]. The treatment of these patients is demanding, both in regards of classification, timing and the surgical set-up.

We present a case of a severe trauma inducing an advanced flail chest with costal fractures and sterno-costal dehiscence, which was treated surgically.

The work has been reported in line with the latest Surgical Case Report (SCARE) criteria [10].

## 2 Presentation of case

**Fig. 1:**
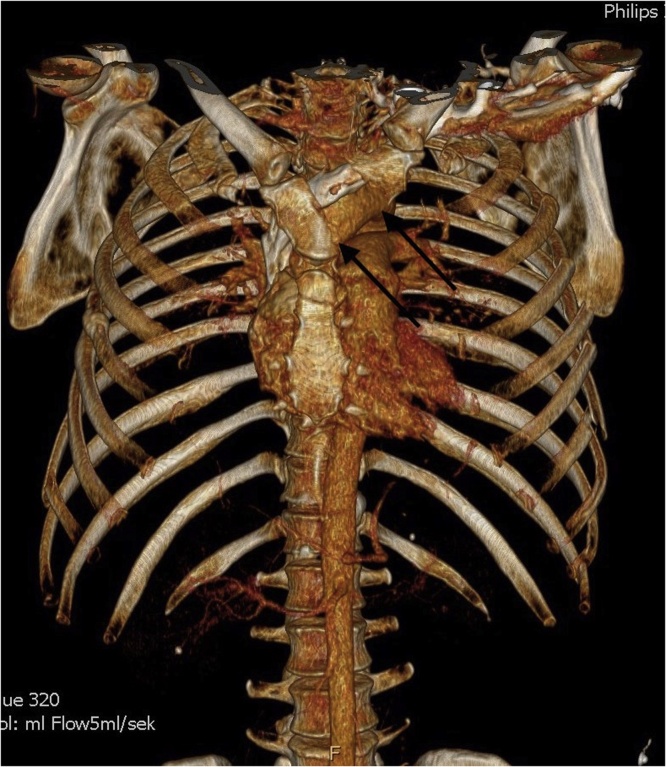
3D reconstruction of the trauma scan showing flail chest with the fracture line anteriorly and several lateral costal fractures on the left side. The arrows are marking the manubrium fracture.

A previously healthy 48-year-old man was involved in a high-speed frontal car accident. The patient was initially unstable and intubated prehospitally. He was brought to the Emergency Room (ER) at a University Hospital, with circulatory distress, but was stabilized by infusion of fluid and blood transfusion. The patient had clinical flail chest, and a trauma CT scan was performed showing multiple costal fractures, sternal and manubrium fracture (Fig. 1). Furthermore, the patient had bilateral lung contusions. Further injuries were fracture of the left tibia and left calcaneus.

**Fig. 2:**
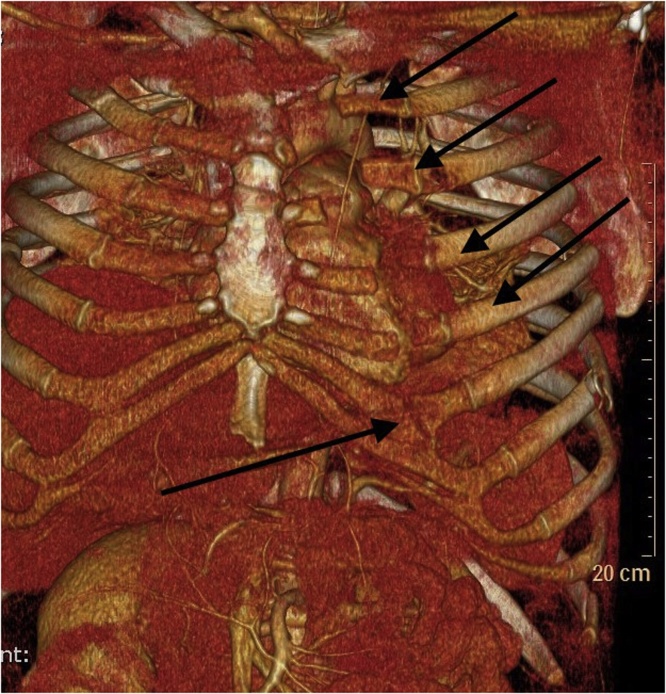
The same trauma scan, but with a 3D reconstruction visualizing cartilage. The arrows are showing the cartilage fractures/dehiscence.

However, the severity of the trauma was not clearly visible in the first 3D bone reconstruction. A new reconstruction was performed with the same software as 3D bone reconstruction using VRT: Volume Rendering Technique. It has a setting that included softer Hounsfield Unit values than those for the bones, thereby clearly revealing the chest cartilage from the same trauma CT scan. After reconstructing the cartilage structure, the sternal dehiscence was perceptible, which is seen in Fig. 2.

After stabilization, the patient was admitted at the ICU. Three days after admission the tibia fracture was osteosynthesized. At day 6 the patient still needed mechanical ventilation, and a tracheostomy was performed. Due to difficult pain management and continued need for mechanical ventilation, it was decided to surgical stabilize the flail chest.

### 2.1 Surgical procedure

**Fig. 3:**
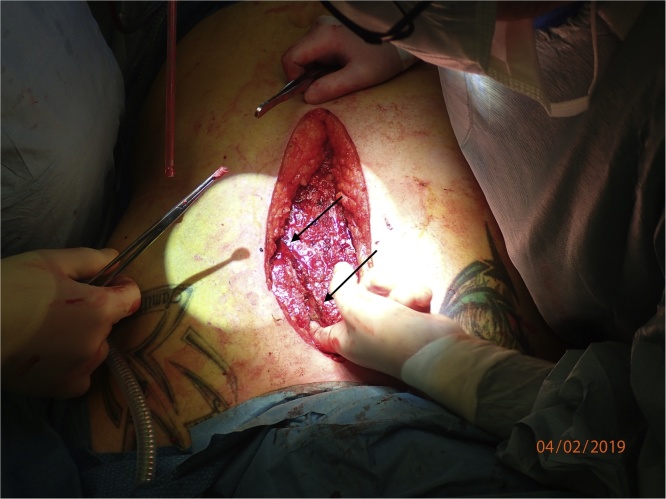
Perioperative picture showing the fractures. The arrows are marking the fracture line.

The patient was placed in a supine position on the back, and the procedure was performed through a midline incision. In order to access the fractures, the left and right pectoral muscles were released of their attachment medially. A localized sub muscular hematoma on the left side was evacuated during the procedure. The osteochondral transitions were fractured with a significant diastase between cartilage and bone (Fig. 3).

**Fig. 4:**
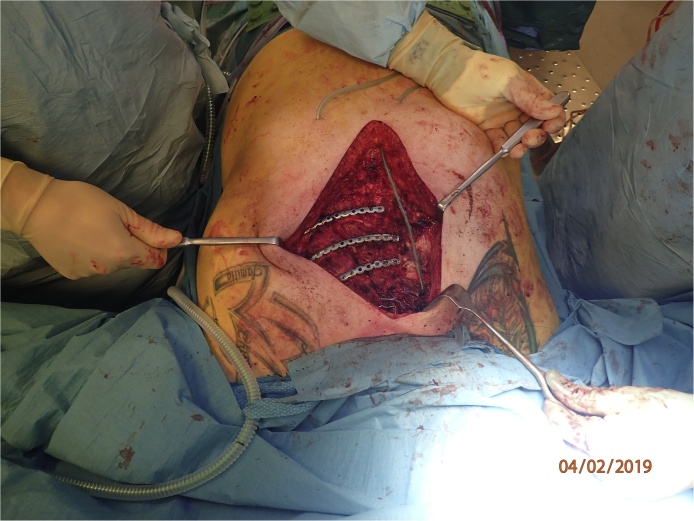
Perioperative picture showing the fixated plates.

A “landing zone” for the plates was prepared on the stenum. The right pectoral muscle was elevated from the ribs to create room for the fixation of the plates. The MatrixRIB™ Fixation System (DePuy Synthes, Johnson & Johnson, USA) was used in this case, where 3 plates were fixated with screws to the sternum and to the adjacent costae (Fig. 4). Finally, the dislocated manubrium fracture was positioned and fixated using 3 cerclage wires, performed as a regular sternal closure following cardiac surgery.

Two handyvac drainage tubes were placed in the sub muscular space. Finally, the pectoral muscle on each side was adapted by suturing the fascia. Hereafter, the wound was closed by suturing the subcutaneous tissue and skin.

The patient was weaned from the mechanical ventilation the first day after surgery, and at day 3 the tracheal tube was removed. At day 4 the patient was moved to the ward for further treatment and rehabilitation, and he was transferred to a local hospital at day 10.

### 2.2 Follow-up

However, the patient was readmitted at our department due to superficial surgical site infection and was treated with Vacuum Assisted Closure (VAC) system (Kinetic Concepts, Inc. (KCI), Acelity™, USA) for 9 days. Hereafter, the defect was closed using syringe, and the patient was discharged from the hospital and went home.

The patient was introduced to a rehabilitation program with a restricted use of the upper body; he was told not pull, push or hang with his arms for the first 6 weeks. After 6 weeks, he was allowed to start using the upper extremities restricted to minimal weight power for additional 6 weeks. During this period, the patient was told to wear a sternal support brace.

**Fig. 5:**
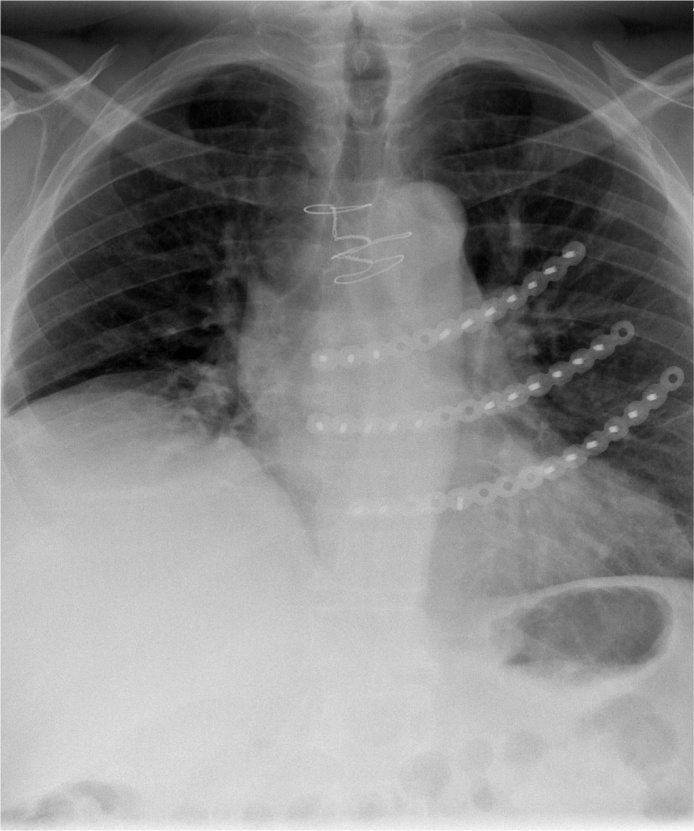
Postoperative chest X-ray visualizing the fixation of the plates to the sternum.

The patient was seen at our outpatient clinic two months postoperatively for follow-up. He had experienced a significant improvement regarding pain level and was no longer using analgesia. He had no signs of surgical site infection, and there was no need for further follow-up. The patient was experiencing trouble with his short-term memory after the trauma, but otherwise well-being. He was able to use his upper extremities without restricted movement, and did not experience breathing problems. Accordingly, there were no longer any restrictions regarding physical activities (Fig. 5).

## 3 Discussion

Severely injured patients will most likely be received or transferred after stabilization to a level 1 trauma center (University Hospital). As part of the trauma evaluation and examination, a CT scan becomes valuable [4]. The trauma CT scan will in most cases reveal a radiological flail chest, if present clinically. However, in this case the trauma scan did not clearly illuminate the full complexity of the trauma before performing a reconstruction including a modality with concomitant visualization of cartilage. Indeed, Fig. 2 clearly reveals the true severity of the trauma, which was not recognized completely by the 3D bone reconstruction displayed in Fig. 1. This indicates, that if a patient has a severe flail chest recognized clinically, but not radiologically, then a reconstruction of cartilage can reveal cartilage fractures with or without sterno-costal dehiscence, which can be missed by a 3D bone reconstruction. Furthermore, the cartilage reconstruction supported the surgeons planning the surgical approach.

The evidence regarding treatment of flail chest is poor and based mainly on small case serie studies. However, these studies indicate that surgical treatment of flail chest is associated with a shorter hospital stay, a reduced need for mechanical ventilation and a reduction of pulmonary complications [6,8,11]. The treatment of patients with flail chest and concomitant sternal fracture is even less evident, due to the very low number of cases. The published cases indicate that such injuries should be treated surgically in order to stabilize thoracic movement, as in patients with flail chest without sternal fracture [9].

In the presented case, the patient experienced a positive outcome from surgery in this case. Already at day 3 after surgery, it was possible to remove the tracheal tube and be transfereed to the ward the following day.

However, the surgical procedure demands correct timing and experience in surgical stabilization of the thoracic wall [9] and requires accurate planning with the involved surgeons and anesthesiologists before surgery.

A recent consensus statement suggests that flail chest patients should be treated surgically within 72 h [12]. In this case, it was not possible to perform the chest wall surgery within 72 h, due to his other injuries which required attention first. All injuries were treated according to their importance.

## 4 Conclusion

Surgical stabilization with costal plates of advanced flail chest with concomitant sternal fracture, sternal dehiscence and costal fractures seems to be a safe procedure, that might reduce the need of mechanical ventilation and the length of stay at the ICU. Furthermore, cartilage reconstruction of the trauma CT scan can potentially identify a severe flail chest, that might not be seen on 3D bone reconstruction.

## Sources of funding

No funding.

## Ethical approval

Since this is a case report, there was no need for ethical approval.

## Consent

Informed consent was obtained from the patient prior to publication.

## Author’s contribution

Ali Imad El-Akkawi: Conceptualization, Methodology, writing – original draft, review and editing. Frank Vincenzo De Paoli: Conceptualization, writing – Original draft, review and editing. Gratien Andersen: Writing – Review and editing Anette Højsgaard: Writing – Review and editing. Thomas Decker Christensen: Conceptualization, Methodology, writing – original draft, review and editing. Supervision and project administration.

## Registration of research studies

No registration needed for this study.

## Guarantor

Thomas Decker Christensen.

## Provenance and peer review

Not commissioned, externally peer-reviewed.

## Declaration of Competing Interest

No conflicts of interest.
